# Editorial: Genetic and Epigenetic Mechanisms of Parkinson's Disease

**DOI:** 10.3389/fnins.2022.842709

**Published:** 2022-04-06

**Authors:** Song Li, Weidong Le, Hao Deng

**Affiliations:** ^1^Center for Clinical Research on Neurological Diseases, The First Affiliated Hospital, Dalian Medical University, Dalian, China; ^2^Center for Experimental Medicine, The Third Xiangya Hospital, Central South University, Changsha, China; ^3^Disease Genome Research Center, Central South University, Changsha, China; ^4^Department of Neurology, The Third Xiangya Hospital, Central South University, Changsha, China

**Keywords:** genetics, epigenetics, neurodegenerative disease, Parkinson's disease, microRNA

Parkinson's disease (PD) is the second most common neurodegenerative disease characterized by four cardinal motor features of tremor at rest, bradykinesia, rigidity, and postural instability (de Lau and Breteler, 2006; Jankovic, 2008). Since the synuclein alpha gene (*SNCA*) missense mutation was discovered in familial autosomal dominant PD in 1997, the understanding of the genetic basis of PD has greatly developed over decades of research (Polymeropoulos et al., 1997). To date, at least 23 loci and 19 disease-causing genes for Parkinsonism have been reported. Additionally, various association studies have also identified a number of genetic risk loci and sporadic PD phenotype genetic variants (Deng et al., 2018). However, the exact biological functions and pathogenic contributions of these genes to this complex disorder remain unclear. Due to the use of high throughout technology and meta analysis we are able to find more PD-related genes and their networks, which will help better understand the genetic mechanisms of PD.

Besides genetic mutations, growing evidence has suggested that the disease pathogenesis might also be orchestrated by epigenetic mechanisms, which could be primarily implemented by methylation of DNA, histone post-transcriptional modifications, and non-coding RNA effects (Feng et al., 2015). For instance, studies have revealed that upregulated *SNCA* gene expression in PD patients' brains might be caused by promoter demethylation. In addition, not only does α-synuclein itself exert epigenetic properties, but also α-synuclein expression levels could be modulated by microRNAs (miRNAs) (Coppedè, 2012). Increasing studies are exploring the epigenetic basis of PD to provide grounds to examine the potential of an epigenetic modifying strategy to counteract PD.

This Research Topic aimed at publishing high-quality articles to further dissect the complex interactions between the genetic basis and epigenetic biomarkers, lifestyles, and environmental factors in PD, and to ultimately advance research with the potential to characterize individuals at risk and produce novel therapeutic approaches. Through rigorous peer review, we have gathered four articles described as follows.

Chronologically, in the first article “Meta-Analysis of Differentially Expressed Genes in the Substantia Nigra in Parkinson's Disease Supports Phenotype-Specific Transcriptome Changes”, Phung et al. used an approach of meta-analysis of differentially expressed genes (meta-DEGs) to mine the associations of DEGs with disease-related or cell type-enriched genes. The study revealed that downregulated meta-DEGs were overlapped with neuron-enriched genes. Furthermore, meta-DEGs were only significantly enriched in PD-related genes, and the enrichment was merely statistically significant in downregulated DEGs, supporting disease phenotype-specific differences in dysregulated genes in PD.

In the article “Dysregulation of MicroRNAs and PIWI-Interacting RNAs in a *Caenorhabditis elegans* Parkinson's Disease Model Overexpressing Human α-Synuclein and Influence of *tdp-1*”, Shen et al. give a molecular landscape of miRNA and PIWI-interacting RNA (piRNA) dysregulation in a *Caenorhabditis elegans* (*C. elegans*) PD model. The authors supposed that miRNAs and piRNAs might relate to the toxicities of human wild-type α-synuclein overexpressing pan-neuronally (HASN^WT^ OX) and human α-synuclein A53T mutant overexpressing pan-neuronally (HASN^A53T^ OX) in *C. elegans*, and the toxicities might depend on *tdp-1*. The authors then validated the hypothesis by identifying the differentially expressed miRNAs and piRNAs from *C. elegans*, transgenic HASN^WT/A53T^ OX *C. elegans*, and crosses with a *tdp-1* knock-out strain. Through enrichment analysis, they also observed that dysregulated miRNAs/piRNAs and predicted targets might contribute to PD.

Subsequently, the third article, “Cell-Type Specific Changes in DNA Methylation of *SNCA* Intron 1 in Synucleinopathy Brains” by Gu et al. intended to further profile the DNA methylation across the *SNCA* intron 1 CpG island (CGI) in the frontal cortex and neuronal/glia nuclei of PD and dementia with Lewy body (DLB) patients. A significant change of DNA methylation was only shown in neuronal nuclei from PD and glia nuclei from DLB compared to controls. This study was the first to report disease-dependent cell-type-specific differential DNA methylation within *SNCA* intron 1 CGI.

The final article by Strafella et al., entitled “Immune System and Neuroinflammation in Idiopathic Parkinson's Disease: Association Analysis of Genetic Variants and miRNAs Interactions”, investigated the burden of SNPs involved in the immune system and neuroinflammation to the susceptibility and progression of idiopathic PD. Expression quantitative loci (eQTLs) analysis described 12 significant SNPs possibly relating to motor and non-motor PD phenomenology. In addition, 11 PD-associated SNPs mapped to 11 novel susceptibility genes which encode proteins were mainly involved in multiple PD-related signaling pathways. The analysis of miRNA-gene-located SNPs revealed the potential role of miR-499a, miR-196a2, and miR-29a in the regulation of neuroinflammatory and neurodegenerative pathways in PD.

In summary, understanding the genetic and/or epigenetic mechanisms responsible for pathological vulnerability to PD is mandatory for progress in stopping neurodegeneration in PD. By soliciting articles on current work in the field, we hope this research topic will provide the reader with up-to-date resource for deepening the understanding of genetic and epigenetic mechanisms underlying PD and foster discussion and collaboration.

## Author Contributions

SL and HD made the first draft that was adjusted by WL. All authors contributed to the article and approved the submitted version.

## Conflict of Interest

The authors declare that the research was conducted in the absence of any commercial or financial relationships that could be construed as a potential conflict of interest.

## Publisher's Note

All claims expressed in this article are solely those of the authors and do not necessarily represent those of their affiliated organizations, or those of the publisher, the editors and the reviewers. Any product that may be evaluated in this article, or claim that may be made by its manufacturer, is not guaranteed or endorsed by the publisher.

## References

[B1] Coppedè F. (2012). Genetics and epigenetics of Parkinson's disease. Scientific World J. 2012, 489830. 10.1100/2012/489830 22623900 PMC3353471

[B2] de Lau L. M. L. Breteler M. M. B. (2006). Epidemiology of Parkinson's disease. Lancet Neurol. 5, 525–535. 10.1016/S1474-4422(06)70471-9 16713924

[B3] Deng H. Wang P. Jankovic J. (2018). The genetics of Parkinson's disease. Ageing Res. Rev. 42, 72–85. 10.1016/j.arr.2017.12.007 29288112

[B4] Feng Y. Jankovic J. Wu Y. C. (2015). Epigenetic mechanisms in Parkinson's disease. J. Neurol. Sci. 349, 3–9. 10.1016/j.jns.2014.12.017 25553963

[B5] Jankovic J. (2008). Parkinson's disease: clinical features and diagnosis. J. Neurol. Neurosurg. Psychiatry 79, 368–376. 10.1136/jnnp.2007.131045 18344392

[B6] Polymeropoulos M. H. Lavedan C. Leroy E. Ide S. E. Dehejia A. Dutra A.. (1997). Mutation in the α-synuclein gene identified in families with Parkinson's disease. Science. 276, 2045–2047. 10.1126/science.276.5321.2045 9197268

